# Defining the light emitting area for displays in the unipolar regime of highly efficient light emitting transistors

**DOI:** 10.1038/srep08818

**Published:** 2015-03-06

**Authors:** Mujeeb Ullah, Ardalan Armin, Kristen Tandy, Soniya D. Yambem, Paul L. Burn, Paul Meredith, Ebinazar B. Namdas

**Affiliations:** 1 Centre for Organic Photonics & Electronics, The University of Queensland, Australia

## Abstract

Light-emitting field effect transistors (LEFETs) are an emerging class of multifunctional optoelectronic devices. It combines the light emitting function of an OLED with the switching function of a transistor in a single device architecture. The dual functionality of LEFETs has the potential applications in active matrix displays. However, the key problem of existing LEFETs thus far has been their low EQEs at high brightness, poor ON/OFF and poorly defined light emitting area - a thin emissive zone at the edge of the electrodes. Here we report heterostructure LEFETs based on solution processed unipolar charge transport and an emissive polymer that have an EQE of up to 1% at a brightness of 1350 cd/m^2^, ON/OFF ratio > 10^4^ and a well-defined light emitting zone suitable for display pixel design. We show that a non-planar hole-injecting electrode combined with a semi-transparent electron-injecting electrode enables to achieve high EQE at high brightness and high ON/OFF ratio. Furthermore, we demonstrate that heterostructure LEFETs have a better frequency response (*f_cut-off_* = 2.6 *kHz*) compared to single layer LEFETs. The results presented here therefore are a major step along the pathway towards the realization of LEFETs for display applications.

Solution processed, cost effective organic optoelectronic devices have attracted great interest from the scientific community and industrial manufacturers^1^,^2^,^3^,^4^,^5^,^6^,^7^,^8^,^9^. Organic light emitting diodes (OLEDs) in particular are becoming increasingly employed in displays^2^,^3^,^4^,^5^,^6^,^7^,^8^,^9^. In commercial OLED displays, polycrystalline silicon-based transistor(s) backplanes with high mobility (100–150 cm^2^/Vs)^10^,^11^ are currently employed in order to drive current and modulate light emission from each OLED element. Interestingly, the processing of the polycrystalline silicon backplane is more complicated than the deposition of the organic OLED components. As such it would be advantageous to develop an integrated solution processed, low-cost switching OLED pixel to reduce the complexity of the transistor backplane and the light-emitting architecture as a whole.

Light emitting field effect transistors (LEFETs) are an emerging class of integrated optoelectronic device with dual functionality, i.e., an OLED and a transistor in a single device structure^12^,^13^,^14^,^15^,^16^,^17^,^18^,^19^,^20^,^21^,^22^,^23^,^24^,^25^,^26^. This dual functionality of the LEFETs provides a pathway to more economical display technologies and a potential means to solve the backplane issue. In order to employ LEFETs for display applications, certain prerequisites must be achieved including: (i) high external quantum efficiency (EQE) at high brightness; (ii) low off current to reduce power dissipation in the device; (iii) high switching capability (ON/OFF ratio); (iv) acceptable temporal response (~5 kHz being acceptable)^8^; and (v) a well-defined and spatially stable light emitting area with a sufficient aperture ratio (the aperture ratio defined as ratio between the emissive portion of a pixel to the entire area of the device including driving transistors) for pixel design.

LEFETs reported to date have shown significant improvement in performance but are not yet suitable for display applications^18^,^19^. Notably for ambipolar LEFETs, both electrons and holes are injected and transported in the light-emitting material leading to maximum recombination and hence high EQE (>5%)^18^,^19^. However, this high EQE was obtained at a minimum of the drain current, which in turn means at the lowest brightness. Furthermore, ambipolar LEFETs tended to have low ON/OFF ratios^18^ and the light emission zone occurs in the area between the source and drain electrodes, and moves with changes in the applied biases. The narrow emission zone leads to a poor aperture ratio. These characteristics suggest that ambipolar LEFETs are not the way forward for display applications. In contrast, heterostructure unipolar LEFETs have been shown to overcome the drawbacks of ambipolar LEFETs, such as disentangling the charge transport properties from emissive properties of device by using a bilayer active system. Such unipolar bilayer LEFETs have been reported to have very low off currents leading to high ON/OFF ratios (>10^5^), a high brightness (>9000 cd/m^2^)^22^, and a light emission zone (close to either drain or source electrode). However, the key problem of existing unipolar heterostructure LEFETs thus far has been their low EQEs (<0.2%) at high brightness, and poorly defined light emitting area - a thin emissive zone at the edge of the electrodes.

In this paper, we report unipolar LEFETs based on solution processed charge transport and emissive polymers with an EQE of up to 1% at a brightness of 1350 cd/m^2^, with a well-defined light-emitting zone suitable for display pixel design. We show that a non-planar source-drain electrode design strategy combined with a semi-transparent electron-injecting electrode enables maintenance of a high EQE at high brightness (by a factor of 10, compared to control LEFETs). In addition, full control over the dimensions of the light emitting area, and hence aperture ratio is achieved allowing for simple pixel design. Furthermore, we demonstrate that the LEFETs can operate at a frequency of 2.6 kHz and have a maximum aperture ratio of 24%. This work therefore represents a major step along the pathway towards the realization of LEFETs for display applications.

Fig. 1a shows the structure of the pixelated non-planar light-emitting transistor device (which we term Pix-LET), and the active channel materials used in this study. The devices were fabricated on a highly *n*-doped conducting silicon wafer with a SiO_2_/ poly(methylmethacrylate) (PMMA) gate dielectric layer. The light-emitting layer was Super Yellow (SY), which was chosen as its properties are widely reported and it is routinely used as test material for new architectural concepts in OLEDs and LEFETs. Solution processed poly(2,5- bis(3-tetradecylthiophen-2-yl)thieno[3,2-b]thiophene) (PBTTT) was used as the hole transport layer. For a Pix-LET, the hole and electron injecting electrodes consisted of Au and a semitransparent CAC stack, respectively. For comparison with the Pix-LET architecture we fabricated two control light emitting transistors. The first control device consisted of a conventional non-planar light-emitting transistor (NPLET-Au/Ca) with Au/Ca as the source/drain electrodes. The second device had planar source and drain electrodes of Au and a semitransparent CAC stack electrode (LET-Au/CAC), respectively. All devices had a channel length of 100 μm and channel width of 16 mm. Full details of the fabrication and testing protocols are presented in the Methods section.

Fig. 2a shows the electrical transfer characteristics of a typical Pix-LET. The relevant electrical output characteristics for the device are shown in Fig. S2. Under *p*-type voltage bias, the Pix-LET device demonstrates excellent linear and saturation regimes with current ON/OFF ratios of >10^4^ with little hysteresis. The extracted hole mobility for the saturation regime obtained from the transfer characteristics was 0.004 cm^2^/Vs. The measured hole mobility is higher by a factor of ~10^2^ than Super Yellow-only LEFETs^21^ showing that hole transport occurs primarily at the PBTTT/PMMA dielectric interface. The electrical transfer and output characteristics of both control devices are compared in Fig 2a and S2, and it can be seen that both have similar transistor characteristics to that of the Pix-LET, with comparable mobility (See Table 1). The slightly higher current in the NPLET-Au/Ca devices indicates that the resistive nature of the Cs_2_CO_3_ layer of the CAC stack that is in contact with the Super Yellow layer affects the electrical properties of the device.

Fig 2b shows the brightness as a function of gate voltage, and Fig. 2c the corresponding EQE versus gate voltage for the Pix-LET and control devices. The EQE of the Pix-LET device increases with the brightness and reaches 1% at 1350 cd/m^2^. This EQE is an order of magnitude higher than the best performing previously reported LEFETs operating in the unipolar regime and importantly is also achieved at higher brightnesses^20^,^21^. The EQE for both the control devices were also measured and are shown in Fig 2c and Table 1. It can be seen that both devices have lower EQEs when compared to the performance of the Pix-LET. The measured EQE for the control devices (see Table 1) were 0.09% at 1400 cd/m^2^ and 0.45% at 1000 cd/m^2^ for the NPLET-Au/Ca and LET-Au/CAC, respectively.

Fig 3 shows optical images of the devices at a voltage bias of V_g_ = −150 V and Vds = −150 V. For the Pix-LET device, bright yellow-green light was visible to the eye with the emission zone defined by the size of the CAC electrode (Fig 3a). In contrast, the light emission zone from the control LET-Au/CAC device was only partially under the CAC electrode (see Fig 3b), and for the NPLET-Au/Ca device (Fig 3c) emission was only observed at the edge of the Ca electrode. Furthermore the light-emitting zone for the Pix-LET and LET-Au/CAC devices remained underneath the electron-injecting electrode (CAC) and did not spread in the transistor channel. The measured aperture ratio of the Pix-LET device at Vg = −150 V and Vds was 24%, which is close to that of a conventional AMOLED pixel (~34%)^9^. The measured aperture ratios for the control NPLET-Au/Ca and LET-Au/CAC devices were significantly lower at 2.5% and 15%, respectively.

The operating mechanism of the Pix-LET device along with energy levels of the different materials is shown in Fig S3. Under *p*-type bias holes are injected directly into the PBTTT layer [ionization potential (IP) ~ 5.1 eV]^20^,^21^ and subsequently into SY (IP = 5.3 eV)^20^,^21^. Under these conditions holes are the major carrier species in the active channel. The thin CAC stack (work function of Cs_2_CO_3_/Ag ~ 2.3 eV)^21^ injects the electrons into the SY layer (EA = 2.9)^24^. Due to the low electron mobility of the SY film the injected electrons accumulate near the SY/CAC electrode interface and this results in a much higher density of exciton formation and hence light emission directly under the CAC electrode. The higher EQE in the Pix-LET is mainly due to the semi-transparent electrode, which allows greater light output (see Fig S4). Furthermore, the non-planar device geometry in the Pix-LET reduces the contact resistance for the holes and forces the carriers to pass through the emissive layer^20^,^24^ leading to a maximum radiative recombination efficiency of ~38%. The calculated maximum recombination efficiency of the control NPLET-Au/Ca and LET-Au/CAC were ~3% and 17%, respectively (see supplementary Table S2).

To obtain a more complete picture of the light emitting area and underlying physics, we measured magnified optical images as a function of gate voltage and drain current (see Figs S5 and S6) for the Pix-LET. The emission zone in the Pix-LET starts from the outside edge of the CAC electrode and fully spreads inwards until emission occurs from the entire CAC electrode at high current density. These results suggest that: i) hole density increases and extends spatially near the semitransparent CAC electrode as shown in Fig. S3; ii) the CAC electrode enhances electron injection and block the holes. In the Pix-LET device the electron-injecting electrode consists of a resistive 6 nm Cs_2_CO_3_ layer, which is an insulator. Hence, the CAC electrode reduces the electrical benefits, i.e. the contact resistance of the metallic non-planar geometry^20^,^24^ (the hole mobility of the Pix-LET is lower by a factor of 10 than the NPLET-Au/Ca device). However, the non-planar geometry with Cs_2_CO_3_ still provides slightly higher electrical characteristics compared to the planar geometry. This means, that the function of the Cs_2_CO_3_ electrode at the interface with the SY in the Pix-LET is to block holes and improve electron injection; iii) the blocked hole density spread underneath the CAC electrode leads to recombination directly under the CAC electrode; iv) the high EQE of the Pix-LET means there is better hole and electron density balance and efficient recombination (due to the non-planar geometry) compared to control devices.

For display pixel applications, a well-defined and spatially stable light emitting area is necessary. To avoid changes in the emission zone with differing drain current, an appropriate dimension of the CAC electrode must be chosen to fix the light-emitting area and hence the aperture ratio for pixel design. This can be easily achieved by setting the CAC electrode dimension equals to the width of the emission zone at light turn on voltage.

To evaluate the frequency response of the Pix-LET device, we measured the light intensity as a function of gate modulation frequency (at a fixed DC source-drain voltage). The light output intensity of the Pix-LET device appears almost flat up to 2 kHz (see Fig. 4). At a higher gate frequency, the light intensity drops significantly, leading to a cut-off frequency of ≈2.6 kHz at −3 dB. For direct comparison, we have also measured the cut-off frequency from the equivalent OLED and single layer LEFET structures^21^. The cut-off frequency for the single layer LEFET and OLED were 76 Hz and 60 kHz respectively. We define the cut-off frequency as the modulation frequency of the gate voltage at which the light output of the system decreases to −3 dB. The −3 dB point frequency is related to the charge carrier transit (*t_tr_*) time^27^ by 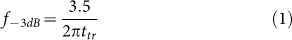
where, the carrier transit time is defined by the applied voltage (*V*), mobility (*μ*) and channel length, *L* (or OLED thickness, *d*). The transit time, 
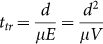
, where, *V* is source-drain voltage in LEFETs (also *Vds* for the transistors) or applied voltage in forward bias for OLEDs. Therefore, the −3 dB frequency can be written as
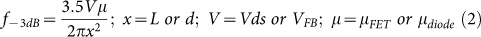
Based on equation (2) and the frequency response obtained from the devices, we can estimate the mobilities to crosscheck with the values obtained from the source-drain current. For the Super Yellow-based OLED with parameters (*d* = 100 nm, *V* = 12 V, *f_-3dB_* = 60 kHz) we obtain a diode mobility of ~8 × 10^−7^ cm^2^/Vs for Super Yellow, which is in agreement with the value estimated from the current and voltage (*V* = 12 V, *j* = 13.5 mA/cm^2^) based on the Mott-Gurney law^28^ - ~3 × 10^−7^ cm^2^/Vs. For the Super Yellow single layer LEFET with *L* = 100 μm, *Vds* = 150 V and *f-_3dB_* = 76 Hz) we obtain an FET mobility of ~9 × 10^−5^ cm^2^/Vs, which is in agreement with steady-state source-drain current measurements^21^. However, the difference in the mobility values from the OLED and LEFET transients is two orders of magnitude. This difference in charge carrier mobilities is due to the charge carrier density. The charge carrier density (Q) in the LEFET channel can be tuned and is a product of the gate voltage (V) and gate capacitance (C) by Q = CV. Thus, the nature of the trap states and trap filling in the bulk (diodes) and at the interface (transistors) is different^29^,^30^,^31^. In the case of the PBTTT/SY bilayer LEFETs using the parameter set *L* = 100 μm, *Vds* = 150 V, and *f−_3dB_* = 2.6 kHz, we obtain a PBTTT FET mobility of 3 × 10^−3^ cm^2^/Vs, which is again in agreement with measured steady-state mobility. These results suggest that the cut-off frequency for the Pix-LET devices is independent of the emissive layer and mainly dependent on the charge transport material and the channel length.

In summary, we have demonstrated a new display pixel design based on bilayer LEFET devices with a transparent drain electrode, which facilitates charge injection and better light out coupling leading to a high external quantum efficiency at usable brightnesses. The device architecture enables decoupling of the low frequency and switching performance of the transistor from the electrical limitations of the emissive material. Our results suggest that the dimension of the CAC electrode and the channel length can be used to set the light-emitting area and hence the aperture ratio for pixel designs. Although the operating voltages of the demonstrated Pix-LETs are still high, these could be reduced by implementing a number of approaches including reducing the channel length and increasing the gate capacitance by employing high k dielectrics or electrolyte gating^32^,^33^. The results are a significant advance towards the ultimate goal of solution processed LEFETs and printed organic semiconductors for display applications.

## Methods

### LEFET fabrication and testing

**Figure 1:**
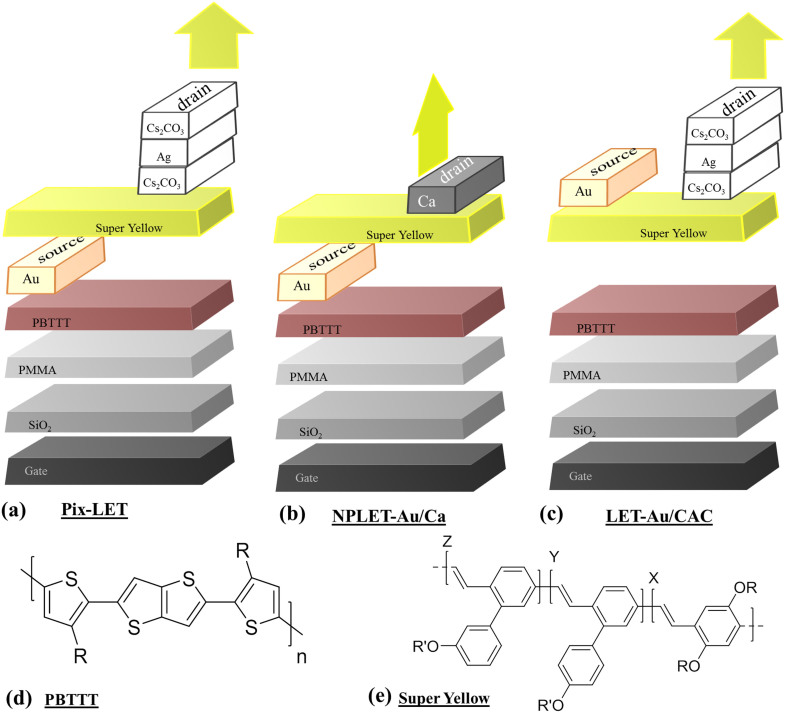
Device structures and materials. Device design of (a) pixelated light-emitting transistor (**Pix-LET**) using a semitransparent drain electrode (b) non-planar light-emitting transistor (**NPLET-Au/Ca**) with conventional Ca-Au drain-source electrodes, and (c) light-emitting transistor in conventional top electrode geometry with a semitransparent CAC drain electrode (**LET-Au/CAC**). Molecular structures of (d) hole transport material PBTTT and (e) of the emissive material Super Yellow.

The hetero-structure LEFETs were fabricated using 300 nm of SiO_2_ and 150 nm of PMMA (Mw ~ 150000) as the gate dielectric layer on a highly *n*-doped silicon wafer as shown in Fig. 1 (a, b and c). Substrates were annealed at 150°C for 30 mins after PMMA deposition and then the hole transport layer of PBTTT (75 nm) was spun on top of PMMA at 1500 rpm for 45 second followed by 2000 rpm for 15 seconds as described earlier^21^. Super Yellow (120 nm) was spin-coated on top of the PBTTT layer from a solution of 7 mg/ml in toluene. All the thicknesses were determined by a Veeco Dektak 150 profilometer. Two shadow masks were used in combination for defining the source and drain electrodes, which were deposited by thermal evaporation in high vacuum to form interdigitated hole-injecting and electron-injecting contacts (see Fig. S1). For the Pix-LET and NPLET-Au/Ca, the hole-injecting electrode “Au” was deposited directly on the top of the PBTTT layer to form a non-planar contact geometry but for the LET-Au/CAC device the Au electrode was deposited on top of the SY film. The electron injecting, semi-transparent Cs_2_CO_3_/Ag/Cs_2_CO_3_ (CAC) stack electrode was deposited on top of the emissive films through successive evaporations of Cs_2_CO_3_, Ag, and Cs_2_CO_3_ at pressure of ~10^−6^ mbar as shown in Fig. 1 (a, b and c) and Fig S1. Thicknesses of 6:10:16 (nm) in the CAC stack were achieved at the evaporation rates of 0.5 A°/s, 1 A°/s and 0.5 A°/s, respectively. The CAC stacks had average sheet resistances of < 8 Ω/□. The sheet resistance for the CAC film was measured using a four-point probe meter from Keithlink while the transmittances were recorded using a UV-vis-NIR spectrophotometer (Cary 5000). For the NPLET-Ca/Au device an 80 nm Ca electrode was evaporated for electron injection instead of CAC.

Electrical and optical characterization of the devices was achieved using an Agilent B1500A Semiconductor Device Analyzer and an SA-6 Semi-Auto Probe station with a calibrated photomultiplier tube (pmt) positioned over the device. The source-drain current in the transistor channel and photocurrent in the pmt were recorded to determine the device parameters. The charge carrier mobility and threshold voltage were calculated from the transfer characteristics in the saturation regime, using equation (3).
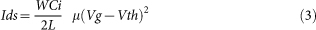
where *Ids* is the source-drain current, *W* is the channel width, *L* is the channel length, *μ* is the field-effect mobility, *C*_i_ is the geometric capacitance of the dielectric, *Vg* is the gate voltage, and *V_th_* is the threshold voltage. The capacitance of the SiO_2_/PMMA dielectric layer was estimated by adding the capacitance of the two layers in series.

The brightness of the devices was calculated from the photocurrent measured with the pmt by comparing with an OLED of known brightness and light emission area, and then corrected according to the measured emission area of the LEFET. A digital camera connected to an optical microscope was used to image the device emission area. The image was then analyzed by taking an intensity profile across the emission region to calculate the width of the emission zone. This was estimated by taking the full-width at half-maximum of the image intensity profile. The EQE was calculated (assuming Lambertian emission) using the brightness, source-drain current and emission spectrum of the device as previously reported^20^,^21^,^22^,^23^,^24^. Averages were taken for at least 5 devices. Errors given are the standard deviation of the results.

### SY OLED fabrication

Glass substrates with pre-etched ITO were purchased from Xinyan Technology Ltd and cleaned by using a soft cloth in a 90°C warm Alconox (detergent) solution. Cleaning was followed by sequential ultrasonication in Alconox, de-ionized water, acetone, and 2-propanol for 15 mins each. After drying the substrates under a nitrogen flow, a poly(3,4-ethylenedioxythiophene):poly(styrene sulfonate) (PEDOT:PSS) (Baytron P VPAl4083) film was spin-coated at 5000 rpm. The resulting 30 nm thick layer was baked at 125°C for 30 minutes in air. All the device edges were cleaned with a wet cloth to prevent current leakage. A solution of Super Yellow was prepared in toluene at 50°C at a concentration of 7 mg/ml. Super Yellow films were prepared by spin-coating at a spin speed of 3000 rpm. The thickness was ~100 nm as determined by a Veeco Dektak 150 profilometer. Finally, 6 nm of barium followed by 100 nm of aluminium was thermally evaporated under a vacuum of 10^−6^ mbar to complete the devices. The resulting device area were 0.2 cm^2^ with 6 devices per substrate.

### OLED frequency test

The OLED voltage was modulated using an Agilent 33250A function generator connected to a voltage amplifier. The OLED light signal was measured using a GaP detector (Thorlab) and an SR530 lock-in amplifier. The OLED was biased at 12 V resulting in a current of 13 mA/cm^2^.

### LEFET frequency test

The gate voltage was modulated using Agilent 33250A function generator connected to a voltage amplifier. The source drain electrodes were biased using an Agilent B1500A Semiconductor Device Analyzer. The output light was measured using a photo-multiplier tube and a Hamamatsu C6438 current amplifier. The signal was acquired using a LeCroy Waverunner A6200 oscilloscope at load resistance of 50 Ohms.

## Author Contributions

M.U. and E.B.N. developed the concept and designed the experiments. M.U., A.A., K.T. and S.Y. set up the experiments. M.U. fabricated the devices and analyzed the data. E.B.N., M.U., A.A., K.T., P.L.B. and P.M. developed the interpretation of the data. M.U., S.Y. and K.T. optimized the transparent electrodes. M.U. and E.B.N. wrote the manuscript with contributions from other authors notably A.A., K.T., P.L.B., P.M. and S.Y. E.B.N., P.M. and P.L.B. supervised the project.

## Supplementary Material

Supplementary Information Supplementary information

## Figures and Tables

**Figure 2:**
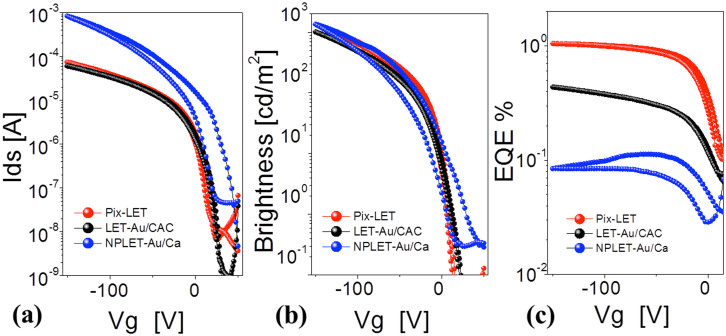
Electrical and Optical Characteristics. (a) Transfer characteristics of the NPLET-Au/Ca, LET-Au/CAC and Pix-LET-Au/CAC devices. (b) Brightness and (c) quantum efficiency of the devices as a function of gate voltage at Vds = −150 V.

**Figure 3:**
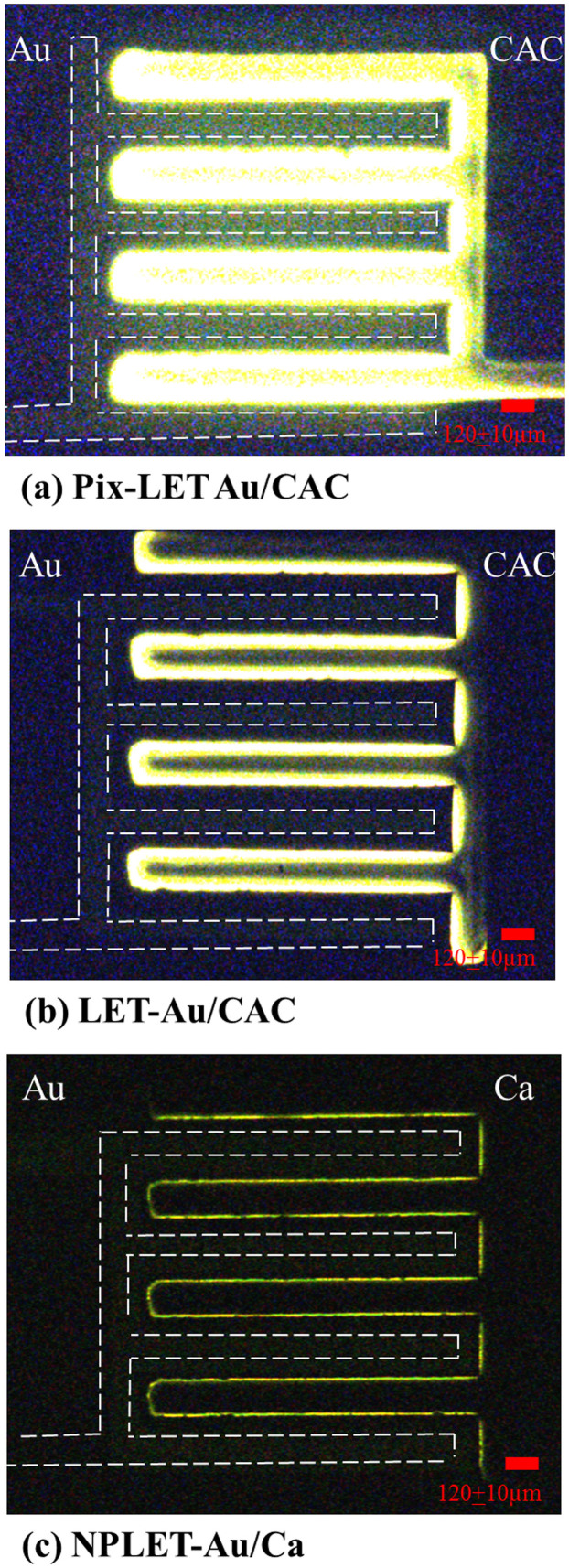
Emission at Vg = −150 V and Vds = −150 V, in (a) Pix-LET, (b) NPLET-Au/Ca, and (c) LET-Au/CAC.

**Figure 4:**
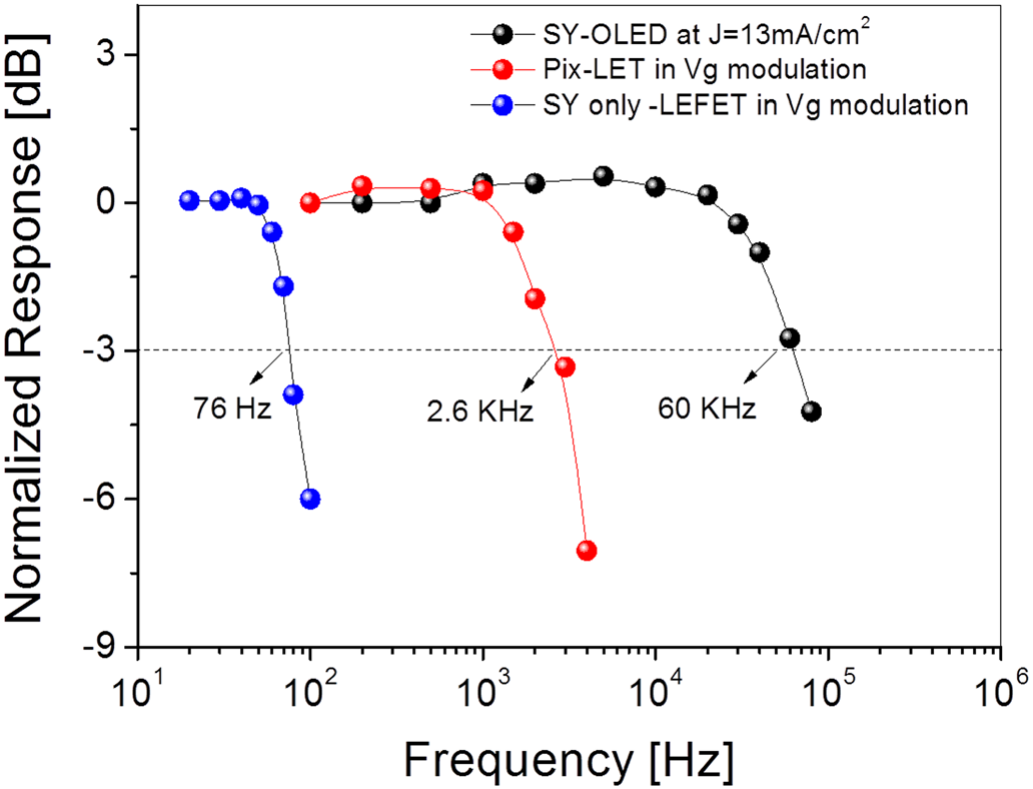
Bode plot for a SY OLED, a 100 μm channel Pix-LET, and a single layer SY-only LEFET^21^ in gate modulation mode.

**Table 1:** Results Summary: Comparison of device results for all three device structures. Averages were taken for at least 5 devices. Errors given are the standard deviation of the results.

	NPLET Au/Ca	LET-Au/CAC	Pix-LET Au/CAC
Device Structure	Bilayer	Bilayer	Bilayer
***μ_h_ [cm^2^/V.s]***	0.08 ± 0.02	0.004 ± 0.001	0.007 ± 0.001
***ON/OFF***	>10^4^	>7 × 10^4^	>10^4^
***Maximum Brightness [cd m^−2^]***	1400 ± 50	1000 ± 100	1350 ± 50
***EQE at maximum brightness [%]***	0.09 ± 0.01	0.45 ± 0.05	1 ± 0.1
***Aperture ratio at Vg = 150 V (light emitting area/total LEFET area)%***	2.5%	15%	24%
