# Supplementary material for: Defining the light emitting area for displays in the unipolar regime of highly efficient light emitting transistors
Source: Sci Rep. 2015 Mar 6;5:8818. doi: 10.1038/srep08818 (PMC4351517; doi:10.1038/srep08818)
Supplement: Supplementary Information [file srep08818-s1.pdf]

Supplementary Information:

Defining the light emitting area for display in the unipolar regime of highly efficient light emitting transistors

*Mujeeb Ullah, Ardalan Armin, Kristen Tandy, Soniya D. Yambem, Paul L. Burn, Paul Meredith, and Ebinazar B. Namdas**

Centre for Organic Photonics & Electronics, The University of Queensland, Australia

*Correspondence to: Ebinazar B. Namdas (e.namdas@uq.edu.au)

Tables S1: Materials, source, and usage.

Figure S1: Fabrication process.

Figure S2: Output characteristics of all the heterostructure LEFETs.

Figure S3: Working mechanism.

Figure S4: Electroluminescence spectrum of the Pix-LET device and transmittance spectrum of the CAC stack electrode.

Figure S5: Emission under the electrodes and not in the channel.

Figure S6: Width of emission zone and drain current for in LEFETs

Table S2: Calculations for Recombination Efficiency

Table S1: Materials, source, and usage.
Material name, description, commercial availability and film deposition method.

Material	Commercial Name	Company	Description	Film deposition	Film Thickness
PBTTT	LISICON 210	Merck	Hole transport	Solution: 4mg/ml in Chlorobenzene	75nm
Super Yellow	Livilux PDY-132	Merck	Yellow emitter, Soluble PPV copolymer	Solution: 7mg/ml in Toluene	120nm
PMMA	Poly(methyl methacrylate)	Sigma Aldrich	Dielectric, surface passivation	Solution: 30mg/ml in propyl acetate	150 nm
Cs_2CO_3	Caesium Carbonate	Sigma Aldrich	Electron injection, hole blocking	Evaporation at rate of 0.5 A°/s	6nm & 16nm
Ag	Silver	Sigma Aldrich	Metal	Evaporation at rate of 1 A°/s	10 nm

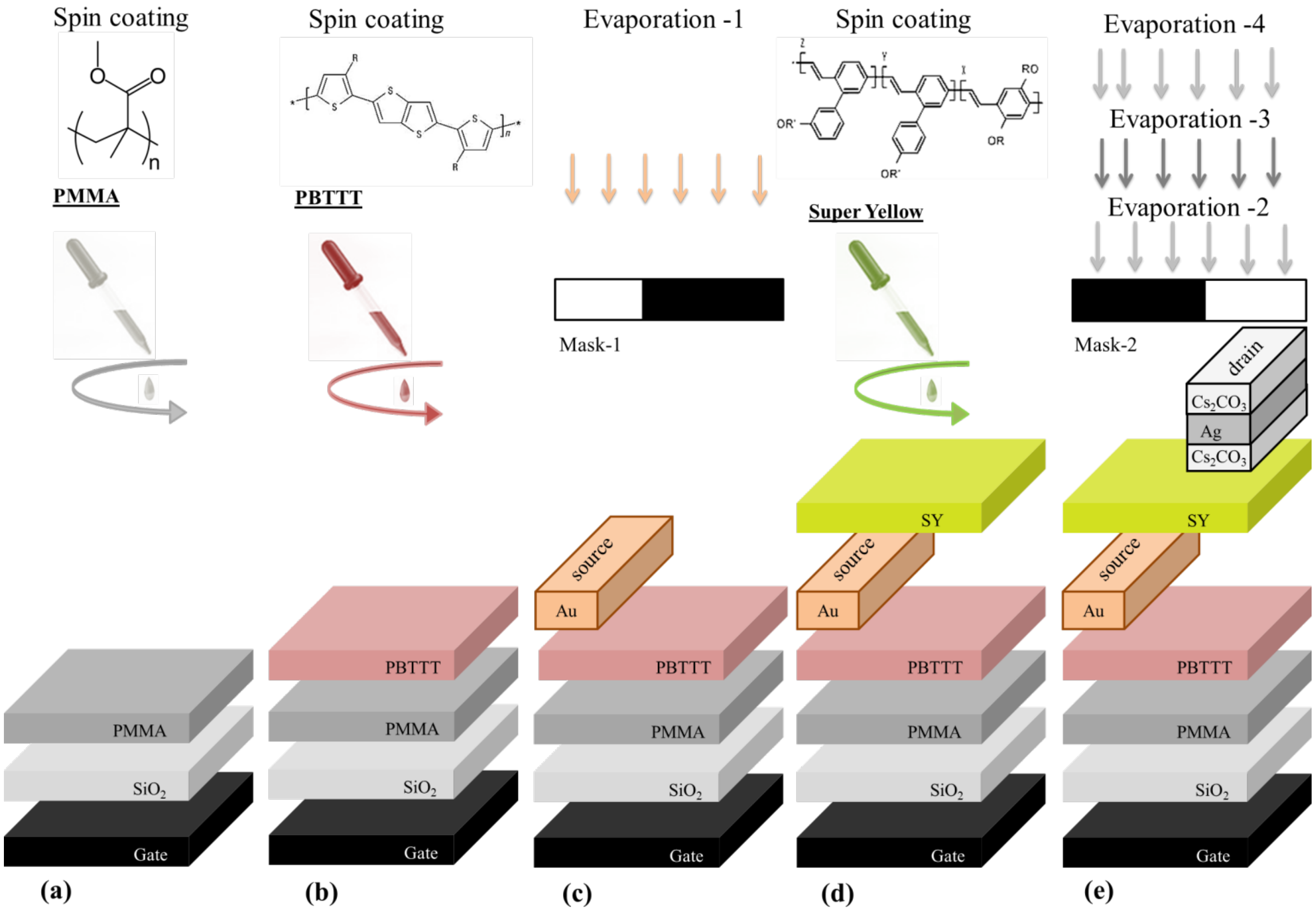

Figure S1: Fabrication Process: Bilayer LEFET devices using **(a)** PMMA passivation layer, **(b)** polymer PBTTT hole transport layer, **(c)** non-planar Au electrode for hole injection, **(d)** Super Yellow emissive layer, and **(e)** top CAC electrode for electron injection and hole blocking.

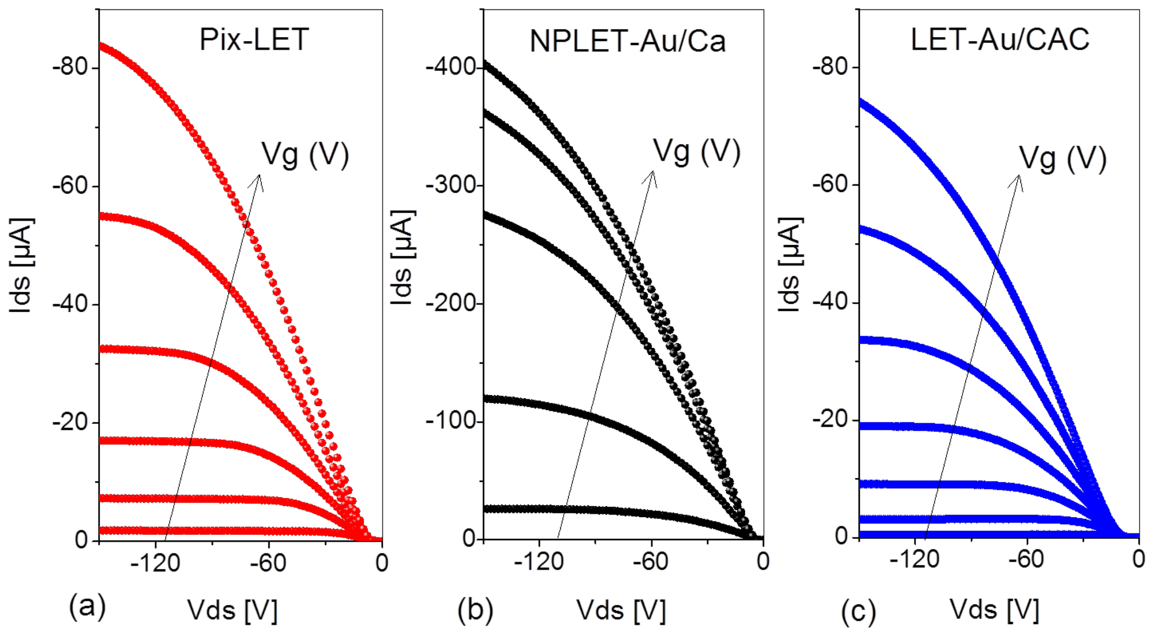

Figure S2: Output characteristics of all the bilayer LEFETs for Vg ranging from 0 V to -150 V, **(a)** Pix-LET **(b)** NPLET-Au/Ca, and **(c)** LET-Au/CAC.

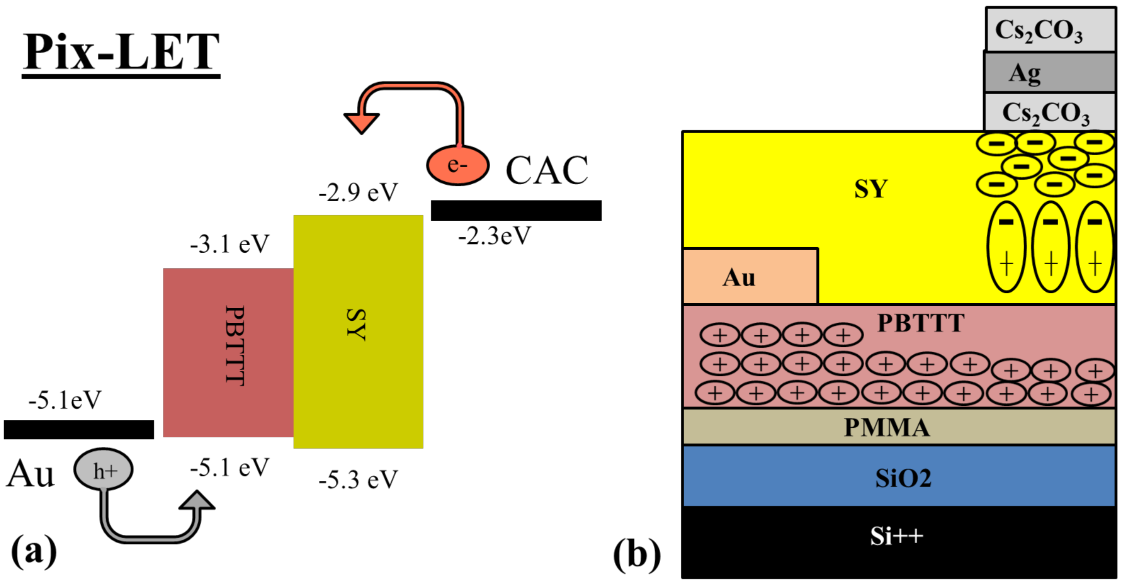

Figure S3: Working mechanism of the Pix-LET-Au/CAC device.

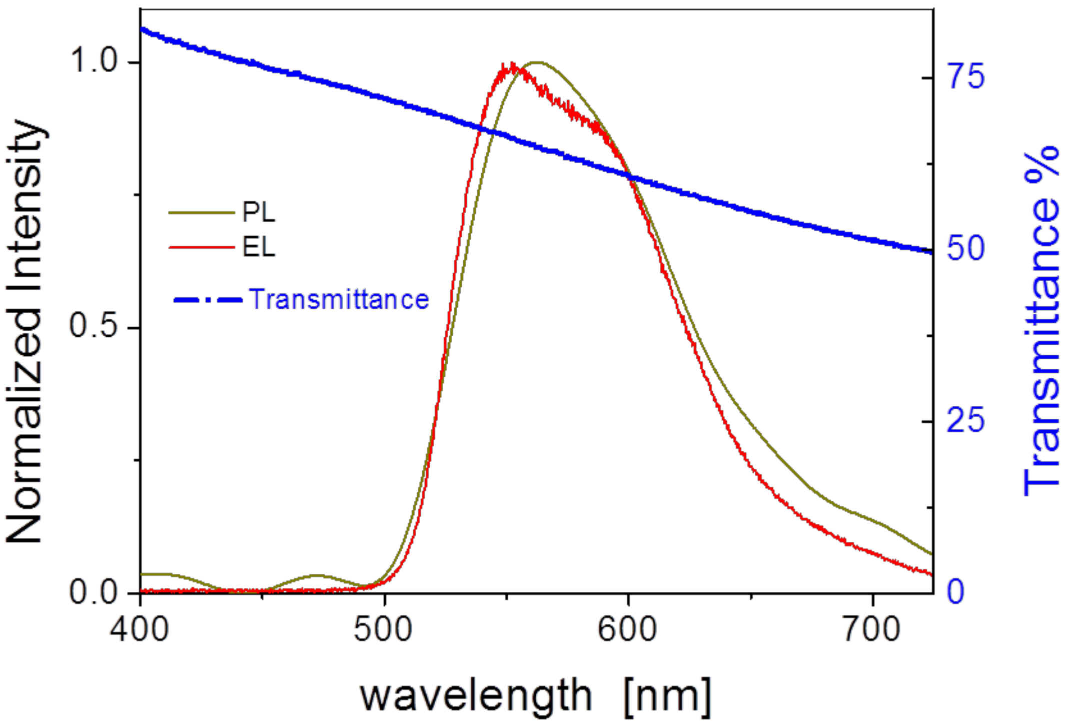

Figure S4 Electroluminescence spectrum of Pix-LET device, and transmittance of CAC stack electrode.

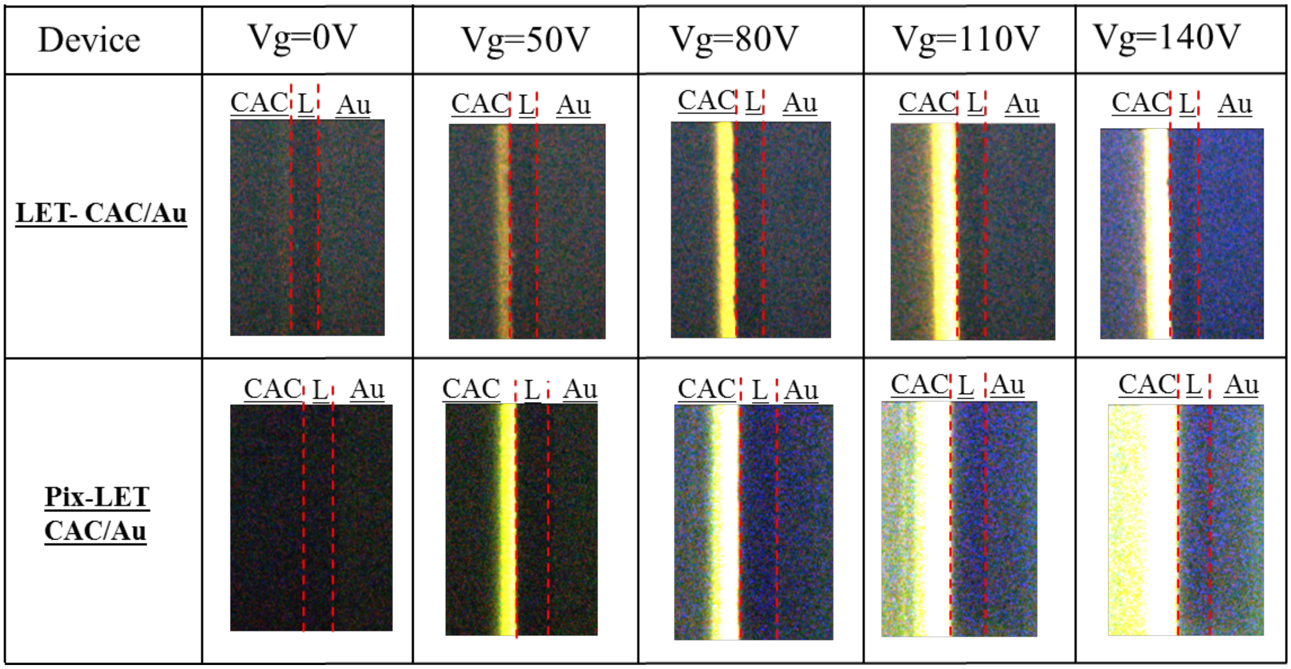

Figure S5: Emission zones for Pix-LET-Au/CAC and LET-CAC/Au. The light-emitting zone remains underneath the electron-injecting electrode (CAC) and do not spread in the transistor channel. The light intensities in the images are not calibrated and uncertainty in the gate voltage is ±10 V.

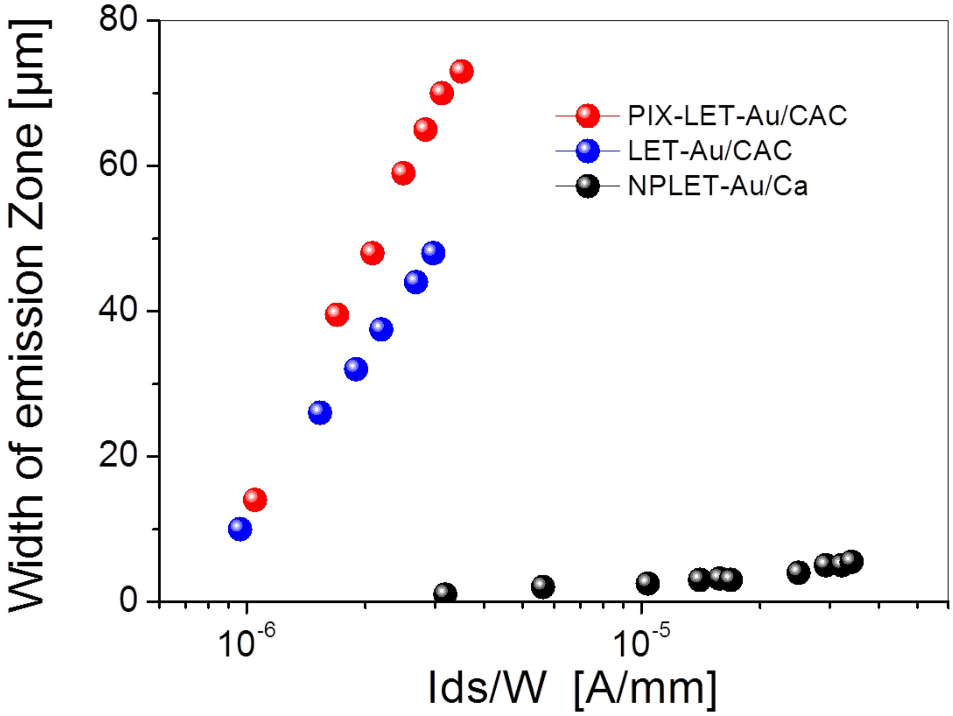

Figure S6: Width of emission zone as a function of drain current density.

Calculating recombination efficiency:

Electrons and the holes in an LEFET channel can form singlet and triplet excitons with the former emissive for fluorescent materials such as Super Yellow. The external quantum efficiency of a device is described by:

$$\phi_{EQE} = \phi_{escape} \times \phi_{capture} \times \phi_{spin} \times \phi_{PLQY}$$

where Φ_{capture} is the fraction of electrons and holes that recombine to form excitons (which is the recombination efficiency), Φ_{EQE} is the calculated EQE, Φ_{escape} is the fraction of photons that can escape from the device, Φ_{spin} is the factor allowed due to spin statistics, and Φ_{PLQY} is the photoluminescence quantum yield (PLQY) in the solid state. The percentage of photons that can escape from the device, Φ_{escape}, is approximately $1/2n^2$ (where n is the refractive index, which for Super Yellow is ≈1.8)[21]. Super Yellow is fluorescent so $\Phi_{\text{spin}} = 0.25$. The solid state PLQY of Super Yellow films prepared under identical conditions was ~68%. Calculated recombination efficiency for all the devices are given below in **Table S2**.

Table S2: Recombination Efficiency

**Device Structure**	**Pix-LET**	**LET-Au/CAC**	**NPLET-Au/Ca**
**EQE [%]**	1 ± 0.1 %	0.45 ± 0.05 %	0.09 ± 0.01 %
**Recombination Efficiency [%]**	38 ± 3.8%	17 ± 1.9 %	3 ± 0.38 %
